# Research trends and highlights in PD-1/PD-L1 inhibitor immunotherapy in lung cancer: a bibliometric analysis

**DOI:** 10.1007/s12672-025-02052-x

**Published:** 2025-03-11

**Authors:** Zheng Gu, Erle Deng, Jing Ai, Fei Wu, Qiang Su, Junxian Yu

**Affiliations:** 1 Department of Pharmacy, Beijing Friendship Hospital, Capital Medical University, Beijing, China; 2 Department of Oncology Center, Beijing Friendship Hospital, Capital Medical University, Beijing, China; 3 National Institute for Nutrition and Health, Chinese Center for Disease Control and Prevention, Beijing, China

**Keywords:** Lung cancer, Immunotherapy, PD-1/PD-L1, Bibliometrics

## Abstract

**Background:**

Lung cancer is one of the most common malignant tumors worldwide. This article aims to review the current research status and trends in PD-1/PD-L1 inhibitor immunotherapy.

**Method:**

On the basis of the Web of Science Core Collection database, literature on PD-1/PD-L1 inhibitor immunotherapy in lung cancer patients was searched and analyzed for all years up to August 5, 2023. Bibliometric techniques were employed, including CiteSpace (6.1.R6), VOSviewer, and the Bibliometrix package in R, to examine publication counts, countries, institutions, authors, journals, cited literature, keywords, and research trends.

**Results:**

A total of 1,252 documents were included following the screening process. The analysis revealed that China had the highest number of publications (512), whereas the institution with the most publications was the UDICE French Association of Research Universities Union (193). The journal with the most articles was the Journal for Immunotherapy of Cancer (48), and the most prolific author was Zhou Caixun from Tongji University in China (20). Co-citation analysis revealed that Borghaei H’s 2015 article in the New England Journal of Medicine had the highest citation frequency. The clustering results indicated that the most frequently referenced keywords included predictors, treatment monitoring, and hyperprogressive diseases. There is a growing trend toward combination therapies, such as dual immune checkpoint inhibitors, and research into molecular mechanisms within the tumor microenvironment, aimed at enhancing the efficacy of immunotherapy and reducing adverse effects.

**Conclusion:**

Bibliometric analysis indicates that PD-1/PD-L1 inhibitors are pivotal in lung cancer immunotherapy. Research in this domain focuses on identifying biomarkers within the tumor microenvironment, addressing immune evasion and resistance to maximize efficacy, and mitigating adverse effects.

## Introduction

Lung cancer is one of the most common malignant tumors worldwide. According to GLOBOCAN 2020,there were an estimated 2.2 million new lung cancer cases (11.4%) and nearly 1.8 million lung cancer deaths (18.0%) in 2020 [1]. The number of male patients significantly exceeded that of female patients. Lung cancer has a poor overall prognosis, with a 5-year survival rate of 18.6% among patients [2]. Lung cancer is classified into two primary types: non-small cell lung cancer (NSCLC) and small cell lung cancer (SCLC). Non-small cell lung cancer (NSCLC) accounts for approximately 80–85% of all cases.

Recent advancements in immunotherapy for cancer and other immunological disorders have been notable, especially with the introduction of immune checkpoint inhibitors (ICIs). These inhibitors block negative regulatory pathways triggered by receptor‒ligand interactions between tumor cells and T cells [3]. Immunotherapy targeting the PD-1/PD-L1 pathway has significantly impacted the treatment of advanced NSCLC.In contemporary approaches, novel agents that modulate the patient's immune system to elicit antitumor responses are being implemented as alternatives to conventional cisplatin chemotherapy. Immunosuppressive pathways, including programmed death receptor 1 (PD-1) and its ligand (PD-L1), have garnered considerable attention. PD-1 is a regulator of T cell growth and activity[4], essential for maintaining immune tolerance and monitoring tumor development[5]. PD-1 induces negative signaling via PD-L1 expression on antigen-presenting cells and tumor cells [6]. Counters-signaling through antigen-specific T-cell receptors (TCRs) and co-stimulatory protein CD28 leads to T cell activation [7], proliferation, and inhibition of cytokine production, ultimately resulting in apoptotic cell death [8]. Inhibition of the PD-1/PD-L1 interaction effectively rescues function of “exhausted” T cells and promotes the activation of antigen-expressing cells [9, 10]. However, the high production costs associated with monoclonal antibodies, insufficient tumor infiltration, and the risk of adverse autoimmune reactions limit their use.

Since 2015, anti-programmed death 1 (PD-1) or anti-programmed death ligand 1 (PD-L1) immunotherapy has become the gold standard for first- or second-line treatment of stage IV NSCLC. Currently, the U.S. Food and Drug Administration has approved the marketing of drugs including nivolumab, pembrolizumab, atezolizumab, durvalumab, cemiplimab and libtayo [11] for the treatment of NSCLC. Nivolumab, pembrolizumab, and cemiplimab are antibodies that target PD-1, whereas atezolizumab and durvalumab target PD-L1. This study explored the key topics in PD-1/PD-L1 immunotherapy for lung cancer through bibliometric analysis, aiming to provide a reference for new drug development and clinical applications.

Bibliometrics quantitatively analyzes the academic literature in terms of volume, citations, relevance, and other indicators to extract meaningful insights into research hotspots and trends. This bibliometric study aims to provide a comprehensive overview of the current knowledge and understanding of PD-1/PD-L1 immunotherapy in lung cancer.

Research has revealed the PD-1/PD-L1 inhibitor immunotherapy research in both clinical and preclinical studies. The therapeutic effect can be enhanced by combining various immunosuppressants, while preclinical investigations focus on reducing adverse reactions and mitigating drug resistance.

## Material and methods

### Sources and collection of information

The Web of Science database is a major source of global academic information**,** covering the natural sciences, engineering technology, biomedicine, social sciences, arts, and humanities. It includes over 13,000 authoritative and high-impact academic journals from around the globe, and is a significant database of global academic information [12]. The core Web of Science Collection was retrieved from a period of all years.

Our retrieval strategy was as follows: “TS = (Pulmonary Neoplasms OR Lung Neoplasm OR Lung Cancer OR Pulmonary Neoplasm OR Pulmonary Cancer OR Cancer of Lung)AND TS = (Anti-PD-1/PD-L1 Immunotherapy OR PD-1/PD-L1 OR PD-1/PD-L1 inhibitor OR PD-1/PD-L1 immunotherapy)”.Choose “article” was chosen for the document type. “English” was selected for the language, and all years were selected for the time range.

Exclusion criteria: The bibliometric analysis included studies focused on PD-1/PD-L1 and lung cancer as primary studies. A total of 604 review papers, 105 conference abstracts, 24 online publications, 22 editorial materials, 12 conference proceedings papers, 11 letters, five book chapters, three revisions, duplicates, and unrelated literature were excluded.

### Methodology

Preliminary bibliometric studies of the raw Web of Science(WOS) data were conducted by exporting the complete records of the literature from the core Web of Science databases, including supplementary references, in plain text format. CiteSpace (version 6.1.R6), VOSviewer, and the R package Bibliometrix were used to analyze the external characteristics of the literature, such as publication year, countries, institutions, authors, journals, citation frequency, keywords, and their clustering. These tools also facilitated a more in-depth analysis of the study content.

## Result

### Overall literature detections

A total of 2016 documents were initially analyzed, resulting in the selection of 1252 relevant documents. This analysis covered 389 journals, 509 authors, 320 institutions, and 59 countries and regions. The literature demonstrated an annual growth rate of 45.07%, with an average citation count of 46.99. The rate of international co-authorship was 21.01%, with a total of 28,483 references and an average of 10.6 co-authors per publication.

### Overview of publication status

**Fig. 1:**
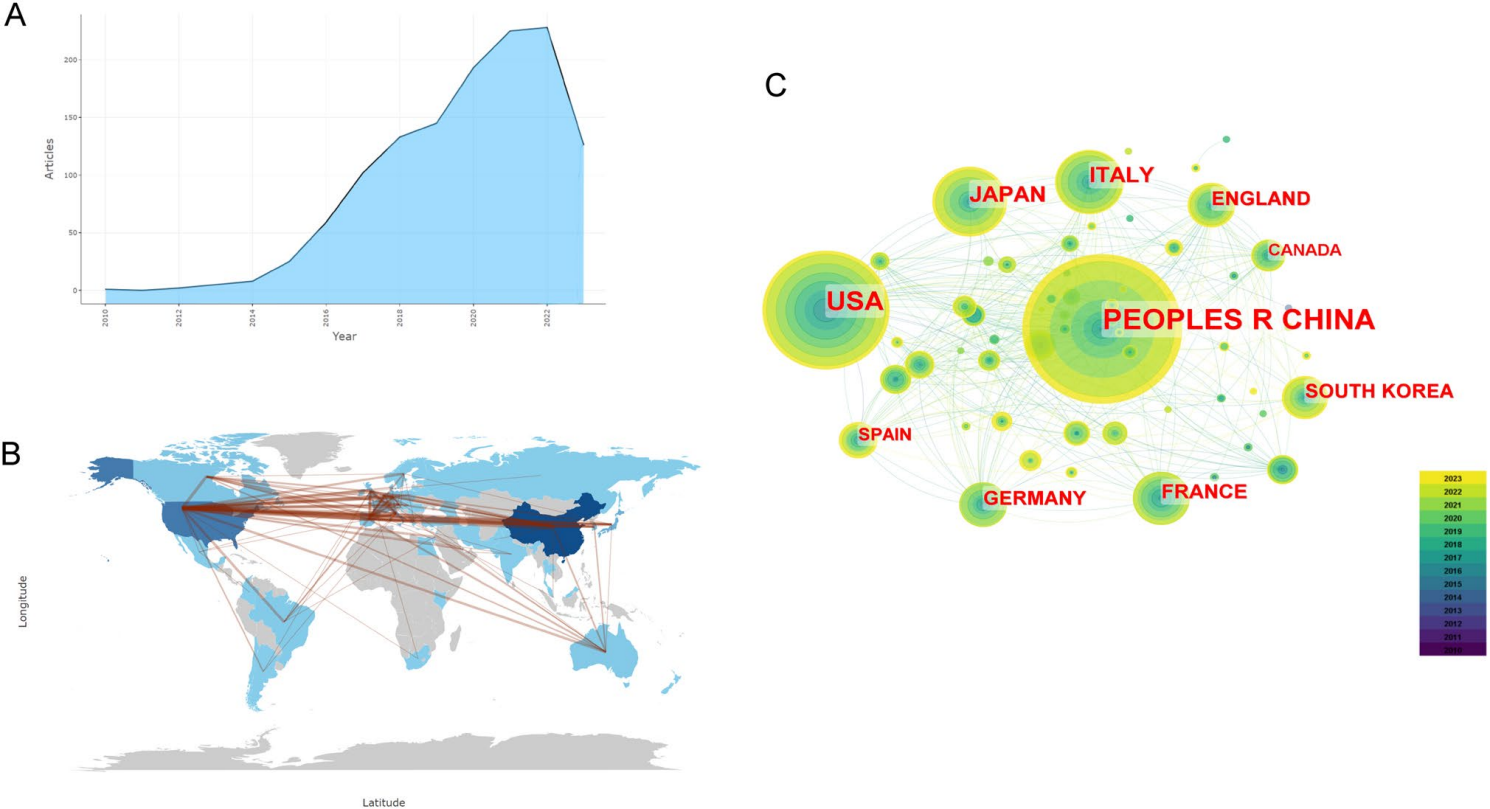
The Overview of Publication Status. **a** Annual Scientific Production trend chart of 2010–2023 **b** Global cooperation network visualization diagram (**c**)Countries in the study visualization map

We compiled all the available literature on PD-1/PD-L1 immunotherapy for lung cancer (Fig. 1A). The study period began in 2010, with a gradual increase in publications over the years. The peak year for publications was 2021, with a total of 107 publications as of August 5, 2023.

### Analysis of national publication counts

Using CiteSpace and the R package Bibliometrix, we generated a visualization map of the study countries (Fig. 1C) and a depiction of the global collaboration networks (Fig. 1B) were generated. A total of 59 countries and territories worldwide were involved in relevant research. China led in the number of publications (publications 512, centrality 0.04), followed by the United States (publications 335, centrality 0.63), which demonstrated the highest centrality, indicating the most extensive collaborative ties with other nations. China**,** however, should strengthen its cooperative efforts with other countries because of its lower centrality. The top ten countries by publication volume were China, the United States, Japan, Italy, France, the United Kingdom, Germany, South Korea, Spain, and Canada. The number of connecting lines signifies the degree of collaboration among nations, with more lines indicating closer cooperation. In the visualization of global collaboration, the countries participating in the research were denoted by the color blue, with darker shades representing higher publication counts.

### Analysis of institution publications

**Fig. 2:**
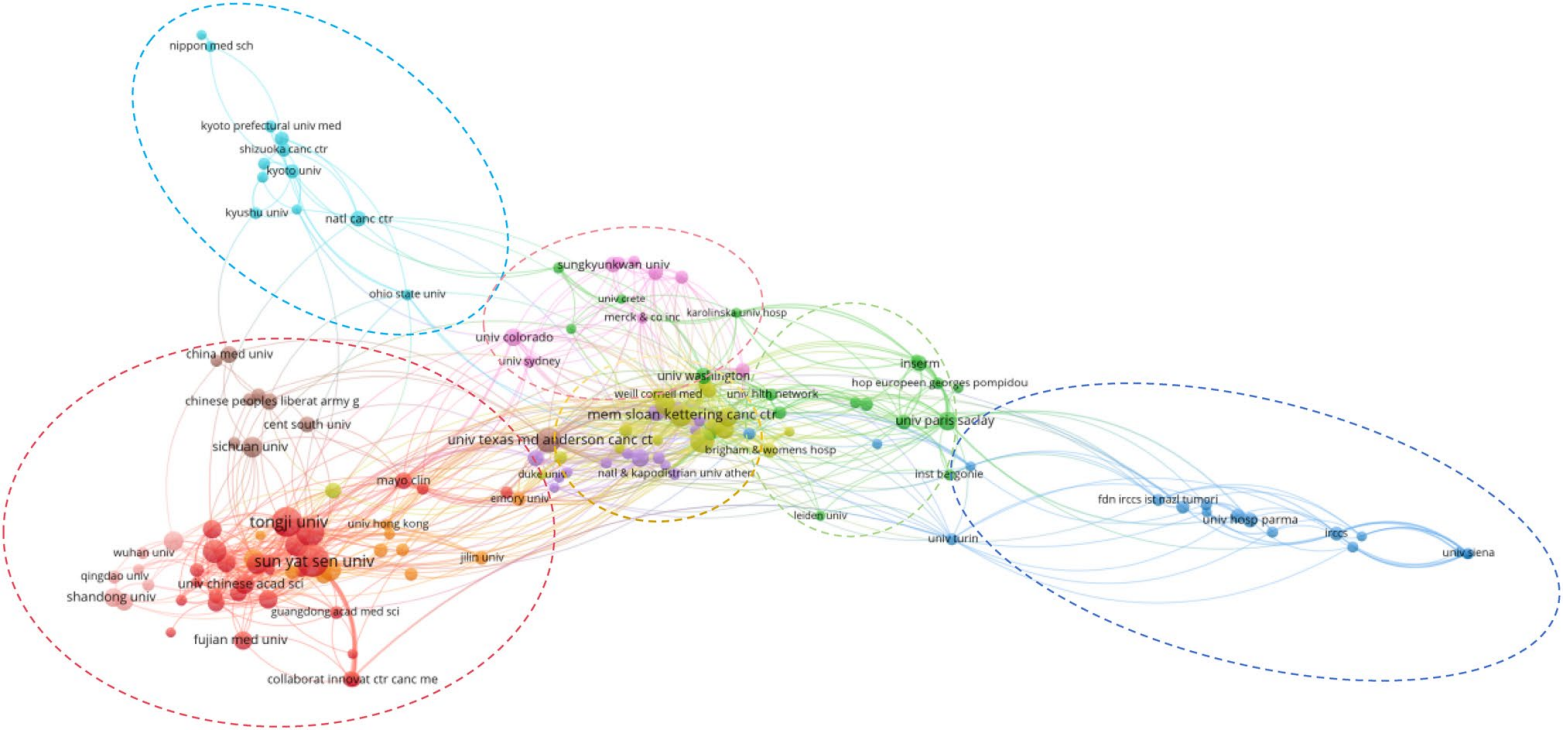
Visual diagram of the research institution

Using VOSviewer, we developed a visual map of research institutions contributing to this field. (Fig. 2). The majority of institutions were located in China, the United States, and France, with the top-ranked institution was France's UDICE French Association of Research Universities Consortium, which accounted for 193 publications. The top 10 institutions were shown in Table 1. The visual map revealed six primary clusters of institutional collaboration. The red cluster represented China, with Tongji University and Sun Yat-sen University in the circle's center. The blue and purple clusters separately represented Japan and America, while green areas indicated European research institutions, and blue regions symbolized Italian institutions.The visualization indicated that research institutions in China contributed significantly to the publication volume. In China, there was a notable degree of collaboration among domestic institutions, though engagement with international entities was comparatively limited. In the visual map, China had more robust relations with the United States, collaborating with partner institutions such as the University of Texas M.D. Anderson Cancer Centre, Harvard Medical School, and Sloan-Kettering Cancer Institute, among others. The University of Paris-Saclay was a central node of French research institutions. The United States engaged in greater collaboration with both Europe and China. The collaborative network maps of Italy and Japan exhibited greater dispersion, revealing a distinctly independent nature within these networks.

**Table 1:** Top 10 institutions in terms of publications

Rank	Institution	Publication	Country	Rank	Institution	Publication	Country
1	UDICE-French Research Universities	193	France	6	UTMD anderson cancer center	86	France
2	Harvard University	142	America	7	Université Paris Cité	77	France
3	UNICANCER	120	America	8	Gustave Roussy	72	France
4	Sun Yat Sen University	112	China	9	UniversitéParis-Saclay	72	France
5	University of Texas System	95	America	10	Chinese Academy of Medical Sciences(CAMS), Peking Union Medical College	71	China

### Analysis of research authors

**Fig. 3:**
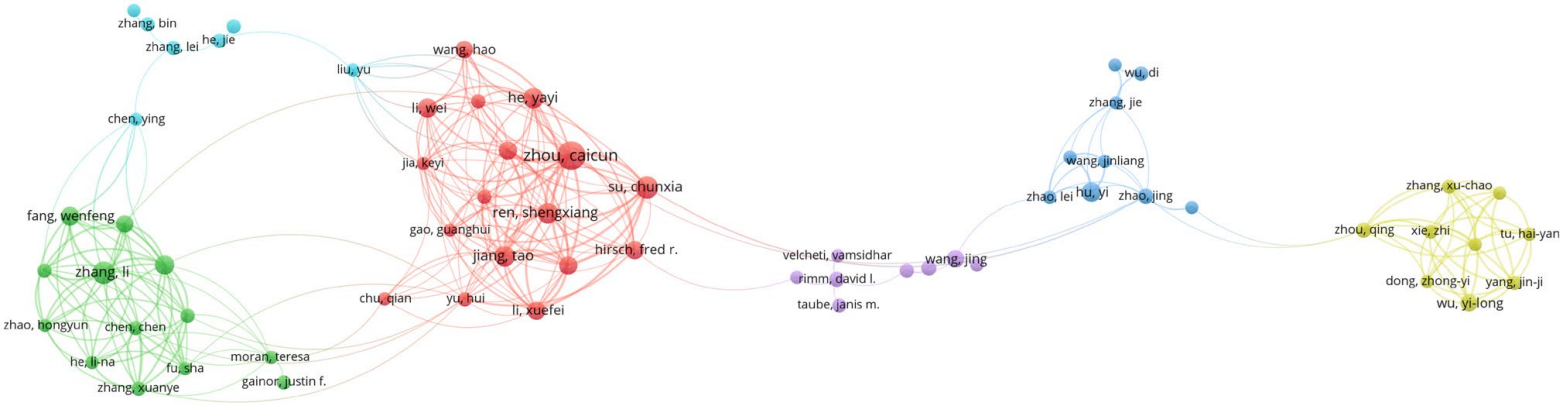
Authors in the publications visualization diagram

Using VOSviewer, we generated the authors’ visualization map (Fig. 3). A total of 509 authors worldwide contributed to this field of study, with Zhou Caicun from Tongji University in China emerging as the most prolific author and having authored 20 publications. Zhou Caicun's research has focused on therapies for EGFR-mutated NSCLC and the tumor microenvironment. Notably, Zhou Caicun's significant study published in the New England Journal of Medicine demonstrated that amivantamab combined with chemotherapy, when used as first-line treatment for patients with advanced non-small cell lung cancer harboring EGFR exon 20 insertions, was more effective than chemotherapy alone [13].

The visualization map indicated six independent research teams from China and America. In China, there were five teams, and the other four teams were separately represented by Hu Yi, Wu Yi-long, Zhang Li, and Zhang Bin. The team led by Zhou Caicun had the most extensive collaborative network and was interconnected with other teams. Wu Yi Long and Zhang Li focused primarily on the clinical trials involving chemotherapy and immunotherapy. While Hu Yi and Zhang Bin's centered their research on the T cell function and immune infiltrating landscape. David Rimm from Yale University led the American team, which consisted of relatively independent scholars. In contrast, the team from Sun Yat-sen University Cancer Prevention and Treatment Center exhibited a more cohesive collaborative network. Research by David Rimm focused on tumor biomarkers for immunotherapy and their bioinformatic analysis.

### Analysis of publication quantity and journal impact

**Fig. 4:**
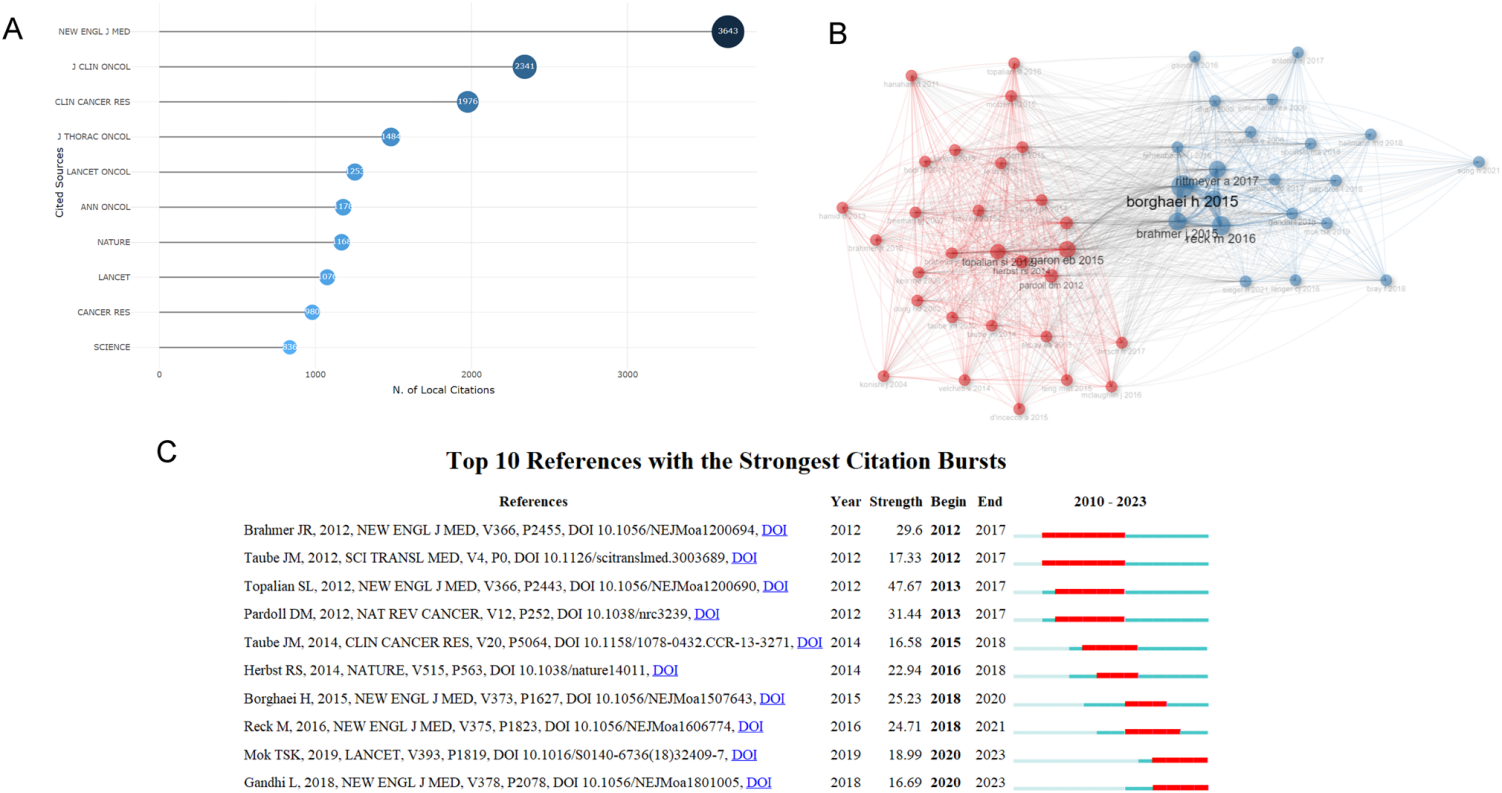
Analysis of journal and co-cited literature. **a** Most Relevant Sources. **b** Co-citation Network. **c** Top 10 references with the strongest citation burst

The 1252 recovered documents were published across 389 journals, with the most relevant sources illustrated in Fig. 4A. The journal with the highest number of publications in this field was JOURNAL FOR IMMUNOTHERAPY OF CANCER,with 48 publications. The journal with the second highest number of publications was FRONTIERS IN ONCOLOGY,with 47 publications. The top 10 journals by publication count, (Table 2**)** include details on the number of publications, impact factor, JCR partition, h-index, g-index, m-index,total citations(TCs), and year of publication start (PY-start).

**Table 2:** Top 10 journals in terms of publications

Rank Journal	Published articles	Proportation	IF	JCR	h-index	g-index	m-index	TC	PY-start
Journal for immunotherapy of cancer	48	3.825%	10.9	Q1	23	47	2.556	2234	2015
Frontiers in oncology	47	3.745%	4.7	Q2	11	15	2.200	327	2019
Cancers	39	3.108%	5.2	Q1	13	21	2.167	486	2018
Lung cancer	37	2.948%	5.3	Q1	18	36	2	1301	2015
Clinical cancer research	33	2.629%	11.5	Q1	26	33	2.6	6485	2014
Oncoimmunology	33	2.629%	7.2	Q1	20	33	2.000	1363	2014
Frontiers in immunology	30	2.390%	7.3	Q1	7	12	1.167	162	2018
Cancer immunology immunotherapy	28	2.231%	5.8	Q1	10	21	1.111	462	2015
Oncotarget	25	1.992%	5.3	Q2	22	25	2	2074	2013
Thoracic cancer	25	1.992%	2.9	Q3	7	14	0.778	226	2015

### Analysis of co-cited literature

**Fig. 5:**
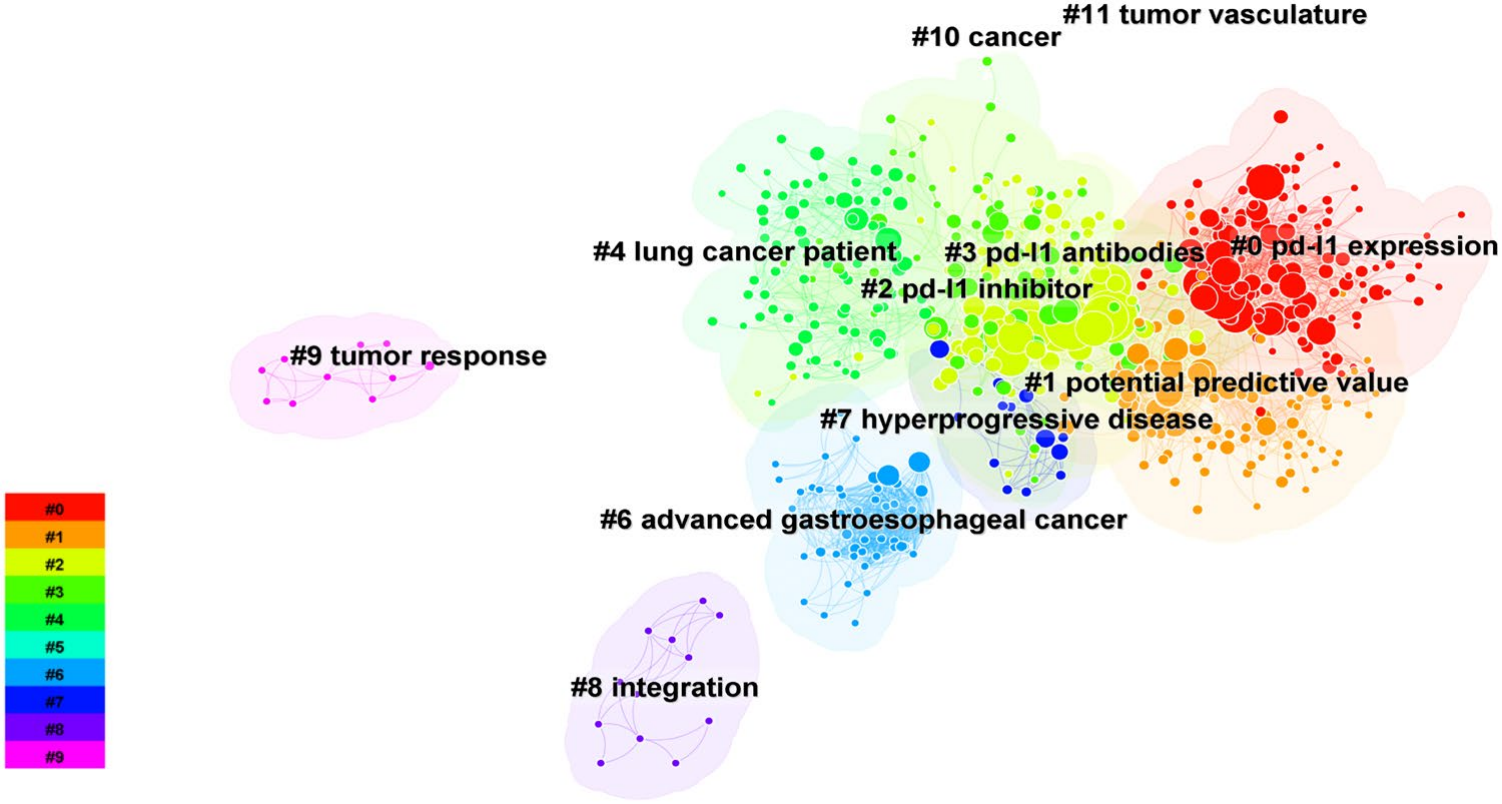
Keywords clustering analysis of the co-cited literature

Using the Bibliometrix package in R, we generated a co-cited visualization map (Fig. 4B), where nodes represent cited literature and connecting lines indicate simultaneous citations between documents. Additionally, keyword clustering analysis of the co-cited literature was performed (Fig. 5). The most frequently cited publication in this field was "Nivolumab versus Docetaxel in Advanced Nonsquamous Non-Small-Cell Lung Cancer" by Borghaei H, published in NEW ENGL J MED, 2015. This study highlighted the improved overall survival associated with nivolumab compared to docetaxel in patients with advanced non-squamous NSCLC who had progressed during or after platinum-based therapy [14]. The keywords from the co-cited literature were clustered and analyzed through CiteSpace, and the results were classified into 11 clusters. CiteSpace cluster analysis of the keywords in the co-cited literature revealed 11 clusters related to PD-L1, non-small-cell lung cancer, chemotherapy, real-world data, predictors, hyperprogressive disease, treatment monitoring, immune‒oncological checkpoints, biogenetics, and molecular imaging.

### Analysis of keyword

**Fig. 6:**
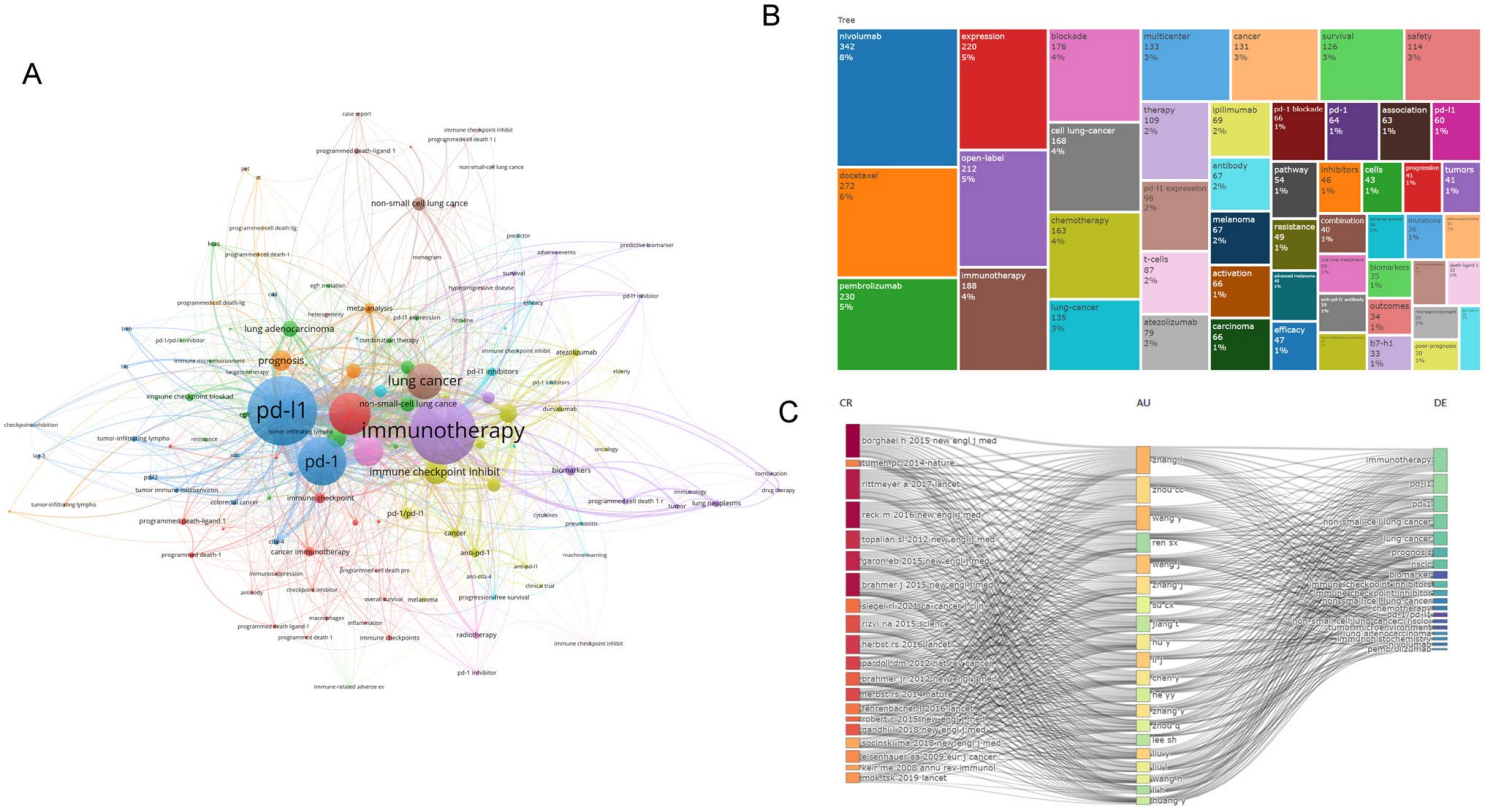
Analysis of keywords. **a** Keywords clustering analysis plot. **b** Keywords visualization map. **c** Author-Reference-Keywords Three-Field Plot

We utilized VOSviewer and CiteSpace for keyword analysis, resulting in the visualization map (Fig. 6**A**). The top 20 keywords identified included nivolumab, docetaxel, pembrolizumab, open-label, cellular lung cancer, chemotherapy, immunotherapy, non-small cell lung cancer, multicenter, PD-L1 expression, treatment, survival, blockade, atezolizumab, T-cells, death ligand 1, safety, ibritumomab, activation, and melanoma. Figure 6B showed the frequency distribution of these keywords, with nivolumab appearing most frequently at 342 occurrences (8%). Author-Cited Literature-Keyword Sankey diagrams were generated to differentiate the research focus areas of various teams (Fig. 6C). Among these, Zhou Caicun's team concentrated on the interaction between tumor microenvironment and immunotherapy, whereas Zhang Li's team focused specifically on the role of pembrolizumab in tumor immunotherapy. The keyword clustering analysis identified 16 clusters, which included themes such as chemotherapy, tumor infiltrating lymphocytes, tumor microenvironment, cancer immunotherapy, non-small cell lung cancer, tumor suppressors, metastatic melanoma, dendritic cells, regulatory T cells, PD-1/PD-L1 inhibitors, lung cancer, immune checkpoint inhibitor, KRAS mutation, immune evasion, neuroendocrine carcinoma, and immune checkpoints.

### Analysis of research trends

**Fig. 7:**
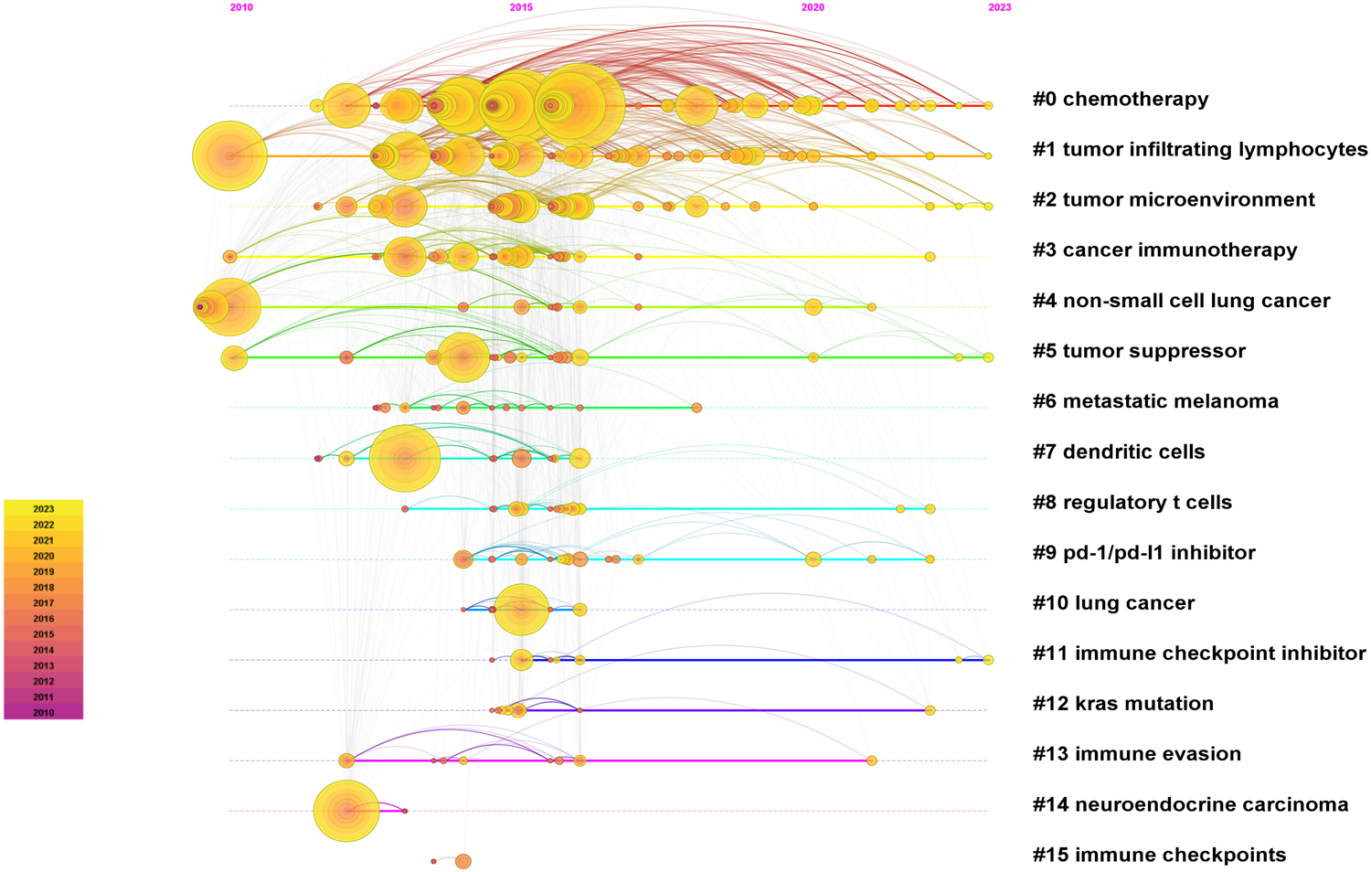
Keywords timeline chart

Using CiteSpace for keyword clustering analysis, we selected the "timeline view" to generate the time-zone map (Fig. 7). As can be seen from the time zone map, the research began in 2010, and the focal keywords from 2010–2015 included tumor-infiltrating lymphocytes, T-cell depletion, chronic viral infection, apoptosis, B7H1 expression, safety, signaling pathways, B7 family, phase I clinical trials, chemotherapy, T-cells and nivolumab. From 2015 to 2020, prominent keywords included phase II clinical trials, metastasis, multicenter, open-label, resistance, immune escape, docetaxel, radiotherapy, atalizumab, biomarkers, randomized controlled trials, EGFR mutations, inflammation, cytokine-induced killer (CIK) cells. For the period from 2020 to 2023, the primary keywords were phase III clinical trials, lymphoid ratio, drug therapy, tumor suppression, tertiary lymphoid structure, combination therapy, toxicity, B-cells, liquid biopsy, immune checkpoint inhibitor ICI, deterioration-free survival, KRAS mutation, dendritic cells, and p53 gene.

## Discussion

This study covered the literature on PD-1/PD-L1 inhibitor immunotherapy for lung cancer over the past fourteen years and analyzed it statistically by publication count, contributing countries, institutions, authors, referenced works, keywords, and research trends. The volume of publications in this field has been steadily increasing, with China and the United States emerging as leaders. China published the greatest number of articles, totaling 512; however, its centrality score of 0.04 indicates limited international collaboration. To strengthen its impact, China should increase collaborative efforts with countries beyond the United States. Most collaborative institutions were based in the United States, reflecting a lack of collaboration among independent research teams in other systems. Chinese universities and research institutions hosted the majority of key authors, with Zhou Caicun from Tongji University in China leading in publication volume with 50 papers. In addition to the systematic research teams that are still limited in other countries, China has developed five relatively independent research teams. Among the top ten institutions, Chinese research institutions demonstrated a stronger trend toward establishing independent teams, contributing to a higher publication output. The journal with the most publications was JOURNAL FOR IMMUNOTHERAPY OF CANCER, and the most cited publication was “Nivolumab versus Docetaxel in Advanced Nonsquamous Non-Small-Cell Lung Cancer” by Borghaei H in NEW ENGL J MED in 2015.

In the last decade, PD-1/PD-L1 immunotherapy has become the predominant treatment modality for stage III-IV non-small cell lung cancer(NSCLC). Compared with alternative ICIs,anti-PD-L1 monoclonal antibodies, including anti-CTLA-4 monoclonal antibodies, have shown prolonged beneficial effects in a diverse array of human cancer patients and exhibit reduced toxicity [15]. Consequently, PD-1/PD-L1 monoclonal antibodies hold significant therapeutic value.

Nivolumab, an anti-human PD-1 monoclonal antibody, was approved for marketing by the FDA on December 22, 2014, and is the first PD-1/PD-L blocker to be used in the second-line treatment of advanced NSCLC [16]. Atezolizumab was approved for marketing by the FDA on May 18, 2016, and pembrolizumab (Keytruda) was approved for marketing by the FDA on September 4, 2014.

The results from the CheekMate017 clinical trial demonstrated that nivolumab significantly improved overall survival (OS), the overall response rate (ORR) and progression-free survival (PFS) [16]. Similarly, the KEYNOTE 010 phase II/III clinical trial, as well as the POPLAR (phase II) and OAK(phase III) clinical trials revealed that the anti-PD-1 antibody pembrolizumab and the anti-PD-L1 monoclonal antibody atezolizumab significantly improved OS, PFS, and ORR in patients with advanced NSCLC compared with docetaxel.

Mutations in oncogenic drivers can affect immune efficacy. A study revealed that among lung cancer patients,37% (36/98) of the samples presented with EGFR mutations, of which 72.2% had exon 19 deletions and 27.8% had exon 21 substitution mutations [17] Mutations in driver genes such as EGFR, ALK, and MET render patients to be sensitive to targeted agents while decreasing their sensitivity to PD-1 monotherapy. However, patients carrying mutations in genes such as PRBM1, TERT, P53, BRCA, and ATM show enhanced responsiveness to PD-1 inhibitor therapy [18, 19]. In 2017, A Grenda et al. analyzed the category of microRNAs associated with PD-L1, which comprised miR-200, miR-197, and miRNA-34, among others. The expression of these molecules could serve as a therapeutic target, qualify for anti-PD-1 or anti-PD-L1 antibody therapy, or aid in diagnosing lung cancer [20]. Investigating biomarkers and other immune factors will influence the effectiveness of immunotherapy. The status of these genes allows for advanced prediction of the patient’s immunotherapy efficacy and further personalized treatment [37]. The results of genetic prediction allow for further personalized treatment.

In 2020, Joseph et al*.* reported that the mechanisms underlying immunotherapy resistance or response can be deciphered by combining mathematical modeling with mechanistic learning algorithms. Increased knowledge of these mechanisms ought to facilitate the identification of implementable components that can augment the effectiveness of immune checkpoint inhibitors [21]. “Genetic magic shears” refers to the capability of CRISPR screening technology to disrupt gene expression and manipulate the genome in the context of immunotherapy resistance research [22]. This method involves screening via genome-wide library-based methods, followed by screening for this resistance gene. Machine learning algorithms have the potential to integrate various genotypes and medications, allowing for the investigation of drug resistance genes and generating predictive insights into their resistance. Expanding the dataset and optimizing the model parameters can improve the prediction accuracy.

In 2021, Edouard et al*.* suggested that mutations in genes such as MET, EGFR, ALK, and BRAF alter the tumor microenvironment, potentially contributing to resistance to anti-PD-1/PD-L1 therapies. In NSCLC with KRAS or BRAF mutations, anti-PD-1/PD-L1 immunotherapy has shown high effectiveness. As more targeted therapies are developed, the inquiry has shifted towards assessing the optimal treatment sequence and combination [11]. Immunotherapy efficacy varies across NSCLC patients with diverse driver gene mutations. A study demonstrated that high TMB and high PD-L1 expression predict clinical benefit in patients with mutation-positive NSCLC driven by ICB therapy. Patients with BRAF-mutated NSCLC may significantly benefit from ICB therapy because of increased TMB and high PD-L1 expression. Despite high PD-L1 expression, NSCLC patients with EGFR and HER2 mutations, and ALK, ROS1, RET, and MET fusions show little benefit from ICB therapy [23].

In 2021, Jaehyun Kim et al. proposed new methods for delivering nanomedicines to specific parts of the tumor microenvironment that can improve immune checkpoint blockade. They also discussed the components and factors within the tumor microenvironment that hinder the effectiveness of these blockades. Understanding the active targeting candidates of components within the tumor microenvironment and the corresponding treatment strategies may offer valuable insights for advancing combination therapeutics that enhance the functionality of immune checkpoint blockades in clinical applications [24].In recent years, researchers have used surface-modified nanoparticles with natural or engineered cell membranes to influence the effects of nanomedicines on tumor immune-related cells. These cell membrane-encapsulated nanomedicines, which have a broad design space and good biocompatibility, have demonstrated obvious advantages in tumor immunotherapy.

In 2022, Martin Reck et al*.* revealed that although the fact that the initial phase of clinical development for immune checkpoint inhibitors, including anti-PD-1 and PD-L1 therapies, primarily focused on second-line monotherapy, recent advancements have introduced combination approaches in first-line settings and integrated immunotherapy into the earlier stages of the clinical paradigm [25].In immunotherapy, cycle duration and withdrawal timing are crucial factors. Treatment typically consists of multiple administrations rather than a single dose, with the timing of cycles tailored to the patient's health status, the disease characteristics, and the physician's treatment plan.

In 2022, Yun Hu et al*.* investigated the integration of NBTXR3-enhanced localized radiation with the concurrent inhibition of three distinct checkpoint receptors—PD1, LAG3, and TIGIT—and evaluated the effectiveness of this treatment was evaluated in a murine model of anti-PD1-resistant lung cancer. The findings decisively supported the efficacy and validity of integrating nanoparticle-enhanced radiotherapy with concurrent inhibition of multiple immune checkpoint receptors. Moreover, they established a preclinical justification for further exploration of this approach in human subjects [26].

The timeline diagram of the keywords reveals three primary phases in PD-1/PD-L1 immunotherapy research: (i)immunotherapy safety studies (2010–2015): During this period, the FDA approved the first PD-1/PD-L1 blockers for advanced NSCLC treatment. The majority of research devoted to the treatment of lung cancer is concerned with first-line therapies such as chemotherapy and related drugs. In contrast, immunotherapy research has focused on the safety, potential immune-related adverse events, and the response and tolerability of monoclonal antibodies in phase I clinical trials. Comprehensive investigations into T-cell mechanisms, including T-cell depletion, are included in this phase. PD-1 is predominantly expressed on T cells, and its interaction with PD-L1 and PD-L2 expressed on antigen-presenting cells(APCs) and tumors transmits inhibitory signals to T cells, potentially leading to T-cell depletion [27]. T cells play crucial roles in the tumor microenvironment, and studies of PD-1/PD-L1 expression mechanisms and signaling pathways have been instrumental in the understanding of immunotherapy. (ii)Research on immunotherapy resistance and biomarkers (2015–2020): During this period, research on PD-1/PD-L1 immunotherapy focused on phase II clinical trials, primarily multicenter, open-label randomized controlled trials. Studies have shown that methylation at the K162 site of the PD-L1 protein significantly reduces its binding to PD-1, resulting in immunotherapy failure due to drug resistance [21]. Therefore, research into immune evasion and drug resistance mechanisms is crucial for developing more effective treatment approaches.PD-L1 expression and tumor mutation burden (TMB) are commonly used biomarkers for PD-1/PD-L1 inhibitor therapy, and real-time therapeutic monitoring of these markers helps predict the efficacy of immunotherapy in patients with lung cancer. There are now an increased number of biomarkers accessible for research on immunotherapy resistance. Currently, five strategies are being employed to address resistance to immune checkpoint blockade [28]. One effective approach is to combine inhibitors targeting multiple immune checkpoints (double immunotherapy) or to use bispecific antibodies, both of which can help overcome immune checkpoint inhibitor resistance. Other strategies include converting "cold" tumors into "hot" tumors, restoring the INF-γ signaling pathway, and targeting tumors that evade immune detection by altering MHC class I molecules. Additionally, targeting suppressive immune cells, such as myeloid-derived suppressor cells (MDSCs), is crucial, as MDSCs contribute to resistance to anti-PD-1 immunotherapy. Approaches to target MDSCs include their depletion, blocking their recruitment, inhibiting their activity, and preventing their differentiation [28].(iii) Combination therapies and immunotherapy research on molecular mechanisms (2020–2023). Recent research has concentrated on drugs in phase III clinical trials, with ongoing studies examining signaling pathways and immune system interactions. Additional genomic and transcriptome analyses are being conducted to explore signaling pathways and predict the response rates to PD-1/PD-L1 immunotherapies. Advanced algorithms and artificial intelligence are utilized to investigate the relationship between drug resistance and the therapeutic response to immunosuppressants. Furthermore, computer vision has been applied to pathology and medical imaging, expediting the evaluation of immunotherapy efficacy. This approach can expedite assessment of immunotherapy efficacy. Current research trends have focused primarily on phase II/III clinical trials to evaluate drug effectiveness and adverse effects. To optimize patient treatment future research will emphasize drug combinations and address challenges related to drug resistance and immune escape, which reduce drug efficacy. Additionally, efforts will be directed at overcoming immunotherapy-related adverse effects, including hyperprogression, pseudoprogression, and delayed response.

The bibliometric analysis of PD-1/PD-L1 immunotherapy revealed that the keywords used in the last three years have focused on immune escape and resistance, tertiary lymphoid structure, combination therapy, KRAS mutation, the p53 gene, Treg cells, dendritic cells, and hyperprogressive disease. These findings indicate that current research hotspots are focused on understanding molecular mechanisms to improve of immunotherapy efficacy and mitigate adverse effects. Additionally, specific novel cells and factors play essential roles in immunotherapy. Recent studies have highlighted a significant link between p53 and cancer immunotherapy, demonstrating that the presence or absence of p53 in cancer cells affects the immune status of the tumor microenvironment. Dendritic cells are also increasingly recognized as important players in immunotherapy. Toll-like receptors (TLRs) play a unique role in regulating the maturation and activity of dendritic cells. They are located intracellularly or on the surface of immune cells and are responsible for recognizing signals from pathogens and tumor cells to activate immune responses [29]. Bin Liu et al. constructed engineered dendritic cells (CXCL9/10-DC) expressing CXCL9 and CXCL10 via lentiviral vectors. These modified dendritic cells enhanced T-cell infiltration and activation within the tumor microenvironment, effectively inhibiting tumor progression in a mouse model of non-small-cell lung cancer. The combination of intratumoral injection of CXCL9/10-DC with immune checkpoint blockade (ICB) therapy effectively overcame therapeutic resistance and established systemic,tumor-specific immunity [30]. However, most patients do not respond optimally to currently approved monotherapies and combination therapies because of immune escape-resistant tumors. This highlights the need for novel immunotherapeutic targets and therapies to improve outcomes for cancer patients. Treg cells in the tumor microenvironment are expected to be potential biomarkers for PD-1 blockade therapy, as they help regulate the immune response in tumors [31]. Real-time therapeutic monitoring is essential for predicting lung cancer immunotherapy outcomes. Moreover, genetic mutations can impact the therapeutic efficacy of PD-1/PD-L1 inhibitors. Multiple clinical studies have demonstrated that the efficiency of PD-1/PD-L1 monoclonal antibodies is only 3% to 7% in NSCLC patients with EGFR or ALK mutations [32], which is notably lower than that of targeted therapy. However, certain mutations, such as those at the p53 locus [33]and kras mutations [34], have been shown to increase the efficacy of PD-1/PD-L1 monoclonal antibodies. Through the investigation of tumor immune mechanisms, novel immunotherapeutic approaches have been developed.

A study [35] revealed that anti-CTLA4 monoclonal antibodies (mAbs) plus anti-PD-1 mAbs appeared to be more effective than anti-PD-L1 mAb therapy in a clinical trial involving NSCLC patients treated with nivolumab and the anti-CTLA4 mAb ipilimumab as part of a dual immune checkpoint blockade [35]. Researchers are actively exploring novel combination therapies based on PD-1/PD-L1 blockade, aiming to improve patient outcomes and increase survival rates. In 2017, Champion et al. [36] introduced the term “hyperprogression” to describe the phenomenon of accelerated tumor progression following immunotherapy. This term may affect the prognosis of immunotherapy-treated patients, thereby diminishing the quality of survival. In this context, a new evaluation framework is needed to establish criteria for assessing the efficacy of treatments for solid tumors. It is essential to consider both short-term tumor remission and the patient's long-term survival benefit, which can be evaluated through a combination of imaging, clinical, serologic, and pathologic assessments. Identifying clinical predictors can help anticipate outcomes, thereby enhancing patient prognoses. However, further research is necessary to reduce the incidence of adverse events.

The significance of our study lies in identifying current hotspots and development trends in PD-1/PD-L1 immunotherapy, providing valuable insights for clinical application and new drug development. Our goals are to identify inadequacies in existing immunotherapy treatments, and possible adverse reactions, recognize potential adverse reactions, and identify biomarkers in the tumor microenvironment that contribute to immune evasion and drug resistance to increase treatment effectiveness. Ultimately, we aim to guide future research initiatives in this field.

There are several limitations to this study. First, our study only included only the Web of Science Core Collection (WoSCC), which covers the majority of high-quality research; therefore, the overall trends in our results remain reliable. Second, some newly published articles may not yet be included in the database, potentially causing a slight delay in identifying emerging hotspots. However, this limitation does not affect the general trend observed.

In conclusion, our bibliometric analysis highlights the essential role of PD-1/PD-L1 inhibitor immunotherapy in lung cancer treatment. Research in this field has focused primarily on identifying biomarkers in the tumor microenvironment, overcoming immune escape and resistance to optimize efficacy, and reducing adverse effects. Additionally, the integration of artificial intelligence and advanced algorithms offers new potential for immunotherapy, and advancements in science and technology will further support research on PD-1/PD-L1 inhibitor immunotherapy.

## Data Availability

The datasets generated and analyzed during this study are available from the corresponding author upon reasonable request.

## References

[1] Sung H, Ferlay J, Siegel RL, Laversanne M, Soerjomataram I, Jemal A, et al. Global cancer statistics 2020: GLOBOCAN estimates of incidence and mortality worldwide for 36 cancers in 185 countries. CA Cancer J Clin. 2021;71(3):209–49. 10.3322/caac.21660. 33538338 10.3322/caac.21660

[2] Noone AM, Cronin KA, Altekruse SF, Howlader N, Lewis DR, Petkov VI, et al. Cancer incidence and survival trends by subtype using data from the surveillance epidemiology and end results program, 1992–2013. Cancer Epidemiol Biomarkers Prev. 2017;26(4):632–41. 10.1158/1055-9965.EPI-16-0520. 27956436 10.1158/1055-9965.EPI-16-0520 PMC5380602

[3] Topalian SL, Weiner GJ, Pardoll DM. Cancer immunotherapy comes of age. J Clin Oncol. 2011;29(36):4828–36. 10.1200/JCO.2011.38.0899. 22042955 10.1200/JCO.2011.38.0899 PMC3255990

[4] Mahoney KM, Rennert PD, Freeman GJ. Combination cancer immunotherapy and new immunomodulatory targets. Nat Rev Drug Discov. 2015;14(8):561–84. 10.1038/nrd4591. 26228759 10.1038/nrd4591

[5] Lesokhin AM, Callahan MK, Postow MA, Wolchok JD. On being less tolerant: enhanced cancer immunosurveillance enabled by targeting checkpoints and agonists of T cell activation. Sci Transl Med. 2015;7(280):280. 10.1126/scitranslmed.3010274. 10.1126/scitranslmed.3010274 25810313

[6] Wang X, Teng F, Kong L, Yu J. PD-L1 expression in human cancers and its association with clinical outcomes. Onco Targets Ther. 2016;9:5023–39. 10.2147/OTT.S105862. 27574444 10.2147/OTT.S105862 PMC4990391

[7] Ostrand-Rosenberg S, Horn LA, Haile ST. The programmed death-1 immune-suppressive pathway: barrier to antitumor immunity. J Immunol. 2014;193(8):3835–41. 10.4049/jimmunol.1401572. 25281753 10.4049/jimmunol.1401572 PMC4185425

[8] Ai L, Xu A, Xu J. Roles of PD-1/PD-L1 pathway: signaling, cancer, and beyond. Adv Exp Med Biol. 2020;1248:33–59. 10.1007/978-981-15-3266-5_3. 32185706 10.1007/978-981-15-3266-5_3

[9] Blackburn SD, Shin H, Freeman GJ, Wherry EJ. Selective expansion of a subset of exhausted CD8 T cells by alphaPD-L1 blockade. Proc Natl Acad Sci U S A. 2008;105(39):15016–21. 10.1073/pnas.0801497105. 18809920 10.1073/pnas.0801497105 PMC2567485

[10] Pauken KE, Wherry EJ. Overcoming T cell exhaustion in infection and cancer. Trends Immunol. 2015;36(4):265–76. 10.1016/j.it.2015.02.008. 25797516 10.1016/j.it.2015.02.008 PMC4393798

[11] Dantoing E, Piton N, Salaün M, Thiberville L, Guisier F. Anti-PD1/PD-L1 immunotherapy for non-small cell lung cancer with actionable oncogenic driver mutations. Int J Mol Sci. 2021;22(12):6288. 10.3390/ijms22126288. 34208111 10.3390/ijms22126288 PMC8230861

[12] Singh VK, Singh P, Karmakar M, Leta J, Mayr P. The journal coverage of web of science, Scopus and dimensions: a comparative analysis. Scientometrics. 2021;126:5113–42. 10.1007/s11192-021-03948-5.

[13] Zhou C, Tang KJ, Cho BC, Liu B, Paz-Ares L, Cheng S, et al. Amivantamab plus chemotherapy in NSCLC with EGFR Exon 20 Insertions. N Engl J Med. 2023;389(22):2039–51. 10.1056/NEJMoa2306441. 37870976 10.1056/NEJMoa2306441

[14] Borghaei H, Paz-Ares L, Horn L, Spigel DR, Steins M, Ready NE, et al. Nivolumab versus docetaxel in advanced nonsquamous non-small-cell lung cancer. N Engl J Med. 2015;373(17):1627–39. 10.1056/NEJMoa1507643. 26412456 10.1056/NEJMoa1507643 PMC5705936

[15] Farkona S, Diamandis EP, Blasutig IM. Cancer immunotherapy: the beginning of the end of cancer? BMC Med. 2016;5(14):73. 10.1186/s12916-016-0623-5. 10.1186/s12916-016-0623-5 PMC4858828 27151159

[16] Brahmer J, Reckamp KL, Baas P, Crinò L, Eberhardt WE, Poddubskaya E, et al. Nivolumab versus docetaxel in advanced squamous-cell non-small-cell lung cancer. N Engl J Med. 2015;373(2):123–35. 10.1056/NEJMoa1504627. 26028407 10.1056/NEJMoa1504627 PMC4681400

[17] Fathi Z, Syn NL, Zhou JG, Roudi R. Molecular epidemiology of lung cancer in Iran: implications for drug development and cancer prevention. J Hum Genet. 2018;63(7):783–94. 10.1038/s10038-018-0450-y. 29666465 10.1038/s10038-018-0450-y

[18] Miao D, Margolis CA, Gao W, Voss MH, Li W, Martini DJ, et al. Genomic correlates of response to immune checkpoint therapies in clear cell renal cell carcinoma. Science. 2018;359(6377):801–6. 10.1126/science.aan5951. 29301960 10.1126/science.aan5951 PMC6035749

[19] Zhang K, Hong X, Song Z, Xu Y, Li C, Wang G, et al. Identification of deleterious NOTCH mutation as novel predictor to efficacious immunotherapy in NSCLC. Clin Cancer Res. 2020;26(14):3649–61. 10.1158/1078-0432.CCR-19-3976. 32241817 10.1158/1078-0432.CCR-19-3976

[20] Grenda A, Krawczyk P. New dancing couple: PD-L1 and MicroRNA. Scand J Immunol. 2017;86(3):130–4. 10.1111/sji.12577. 28675453 10.1111/sji.12577

[21] Ciccolini J, Benzekry S, Barlesi F. Deciphering the response and resistance to immune-checkpoint inhibitors in lung cancer with artificial intelligence-based analysis: when PIONeeR meets QUANTIC. Br J Cancer. 2020;123(3):337–8. 10.1038/s41416-020-0918-3. 32541872 10.1038/s41416-020-0918-3 PMC7403333

[22] Liu J, Xing L, Li J, Wen K, Liu N, Liu Y, et al. Epigenetic regulation of CD38/CD48 by KDM6A mediates NK cell response in multiple myeloma. Nat Commun. 2024;15(1):1367. 10.1038/s41467-024-45561-z. 38355622 10.1038/s41467-024-45561-z PMC10866908

[23] Negrao MV, Skoulidis F, Montesion M, Schulze K, Bara I, Shen V, et al. Oncogene-specific differences in tumor mutational burden, PD-L1 expression, and outcomes from immunotherapy in non-small cell lung cancer. J Immunother Cancer. 2021;9(8):e002891. 10.1136/jitc-2021-002891. 34376553 10.1136/jitc-2021-002891 PMC8356172

[24] Kim J, Hong J, Lee J, Fakhraei Lahiji S, Kim YH. Recent advances in tumor microenvironment-targeted nanomedicine delivery approaches to overcome limitations of immune checkpoint blockade-based immunotherapy. J Control Release. 2021;10(332):109–26. 10.1016/j.jconrel.2021.02.002. 10.1016/j.jconrel.2021.02.002 33571549

[25] Reck M, Remon J, Hellmann MD. First-line immunotherapy for non–small-cell lung cancer. J Clin Oncol. 2022;40(6):586–97. 10.1200/JCO.21.01497. 34985920 10.1200/JCO.21.01497

[26] Hu Y, Paris S, Bertolet G, Barsoumian HB, He K, Sezen D, et al. Combining a nanoparticle-mediated immunoradiotherapy with dual blockade of LAG3 and TIGIT improves the treatment efficacy in anti-PD1 resistant lung cancer. J Nanobiotechnology. 2022;20(1):417. 10.1186/s12951-022-01621-4. 36123677 10.1186/s12951-022-01621-4 PMC9484155

[27] Dyck L, Mills KHG. Immune checkpoints and their inhibition in cancer and infectious diseases. Eur J Immunol. 2017;47(5):765–79. 10.1002/eji.201646875. 28393361 10.1002/eji.201646875

[28] Kalbasi A, Ribas A. Tumour-intrinsic resistance to immune checkpoint blockade. Nat Rev Immunol. 2020;20(1):25–39. 10.1038/s41577-019-0218-4. 31570880 10.1038/s41577-019-0218-4 PMC8499690

[29] Sadeghzadeh M, Bornehdeli S, Mohahammadrezakhani H, Abolghasemi M, Poursaei E, Asadi M, Zafari V, et al. Dendritic cell therapy in cancer treatment; the state-of-the-art. Life Sci. 2020;1(254): 117580. 10.1016/j.lfs.2020.117580. 10.1016/j.lfs.2020.117580 32205087

[30] Lim RJ, Salehi-Rad R, Tran LM, Oh MS, Dumitras C, Crosson WP, et al. CXCL9/10-engineered dendritic cells promote T cell activation and enhance immune checkpoint blockade for lung cancer. Cell Rep Med. 2024;5(4): 101479. 10.1016/j.xcrm.2024.101479. 38518770 10.1016/j.xcrm.2024.101479 PMC11031384

[31] Gianchecchi E, Fierabracci A. Inhibitory receptors and pathways of lymphocytes: the role of PD-1 in treg development and their involvement in autoimmunity onset and cancer progression. Front Immunol. 2018;17(9):2374. 10.3389/fimmu.2018.02374. 10.3389/fimmu.2018.02374 PMC6199356 30386337

[32] Gainor JF, Shaw AT, Sequist LV, Fu X, Azzoli CG, Piotrowska Z, et al. EGFR Mutations and ALK rearrangements are associated with low response rates to PD-1 pathway blockade in non-small cell lung cancer: a retrospective analysis. Clin Cancer Res. 2016;22(18):4585–93. 10.1158/1078-0432.CCR-15-3101. 27225694 10.1158/1078-0432.CCR-15-3101 PMC5026567

[33] Wang S, Jiang M, Yang Z, Huang X, Li N. The role of distinct co-mutation patterns with TP53 mutation in immunotherapy for NSCLC. Genes Dis. 2020;9(1):245–51. 10.1016/j.gendis.2020.04.001. 35005121 10.1016/j.gendis.2020.04.001 PMC8720680

[34] Liu C, Zheng S, Wang Z, Wang S, Wang X, Yang L, et al. KRAS-G12D mutation drives immune suppression and the primary resistance of anti-PD-1/PD-L1 immunotherapy in non-small cell lung cancer. Cancer Commun (Lond). 2022;42(9):828–47. 10.1002/cac2.12327. 35811500 10.1002/cac2.12327 PMC9456691

[35] Dougall WC, Roman Aguilera A, Smyth MJ. Retracted: Dual targeting of RANKL and PD-1 with a bispecific antibody improves anti-tumor immunity. Clin Trans Immunol. 2019;8(10):e01081. 10.1002/cti2.1081. 10.1002/cti2.1081 PMC6763724 31572609

[36] Champiat S, Dercle L, Ammari S, Massard C, Hollebecque A, Postel-Vinay S, et al. Hyperprogressive disease is a new pattern of progression in cancer patients treated by Anti-PD-1/PD-L1. Clin Cancer Res. 2017;23(8):1920–8. 10.1158/1078-0432.CCR-16-1741. 27827313 10.1158/1078-0432.CCR-16-1741

[37] Fathi Z, Mousavi SAJ, Roudi R, Ghazi F. Distribution of KRAS, DDR2, and TP53 gene mutations in lung cancer: an analysis of Iranian patients. PLoS ONE. 2018;13(7): e0200633. 10.1371/journal.pone.0200633. 30048458 10.1371/journal.pone.0200633 PMC6061986

